# Gut microbiome of captive wolves is more similar to domestic dogs than wild wolves indicated by metagenomics study

**DOI:** 10.3389/fmicb.2022.1027188

**Published:** 2022-11-01

**Authors:** Lei Chen, Mengyao Sun, Di Xu, Zenghao Gao, Yuying Shi, Shen Wang, Yiping Zhou

**Affiliations:** College of Life Sciences, Qufu Normal University, Qufu, China

**Keywords:** C*anis lupus*, *Canis lupus familiaris*, captivity, gut microbiome, environmental adaption

## Abstract

Adaptation during the domestication from wolves (*Canis lupus*) to dogs (*Canis lupus familiaris)* is a debated ecological topic. Changes in food and environment are major divergences in the domestication of dogs. Gut microbes play an important role in animal adaptation to the food and environmental changes. In this study, shotgun sequencing was performed to compare the species diversity and functional diversity of gut microbes in wild wolves (group CLW, *n* = 3), captive wolves (group CLC, *n* = 4), and domestic dogs (group CLF, *n* = 4). The results found that Bacteroidetes, Firmicutes, Fusobacteria, Proteobacteria and Actinobacteria were the most abundant phyla and *Bacteroides, Fusobacterium, Prevotella, Megamonas, Paraprevotella, Faecalibacterium, Clostridium* were the most abundant genera in the gut of wolves and dogs. Groups CLW, CLC and CLF have shown significant difference in gut microbial species diversity and functional diversity. *Bacteroides*, *Fusobacterium* and *Faecalibacterium* were most abundant genera in groups CLW, CLC and CLF, respectively. Their abundance varied significantly among groups. Compared to the wild wolves, the intestinal microbiol genes of domestic dogs were significantly enriched in the carbohydrate metabolism pathway of KEGG database. One hundred and seventy-seven enzymes were detected with significantly higher abundance in group CLF than that in group CLW, and 49 enzymes showed extremely significant higher abundance in group CLF than that in group CLW (*q* < 0.01) base on the function abundance annotated in CAZy database. It is noteworthy that there were also significant differences in the abundance of 140 enzymes between groups CLC and CLW (*q* < 0.05). Clustering analysis based on both the species and the function abundance of intestinal microbiota all found that groups CLC and CLF clustered into one branch, while samples from group CLW clustered into the other branch. This result suggests that captive wolves are more similar to domestic dogs than wild wolves in both species composition and function composition of intestinal microbiota.

## Introduction

Ecological adaptation in animal domestication is a debated ecological topic in evolutionary ecology. The dog (*Canis lupus familiaris)* is the first known domesticated animal. Genetic, behavioral and morphological studies have shown that the domestic dog is descended from wolves (*Canis lupus*; Wayne, 1993). Changes in food and environment are major divergences between domestic dogs and wolves. During the early evolution of animals, the ability to digest many important nutrients was gradually lost and replaced by symbiotic microbes (Moran, 2006; Ley et al., 2008). These microbes help the host to uptake nutrients, regulate fat storage (Turnbaugh et al., 2006; Greenblum et al., 2012), enhance intestinal immune function (Ganal et al., 2012; Markle et al., 2013), and play an important role in host food and environmental adaptation. The adaptive changes during the domestication from wolves to domestic dogs in digestive physiology had attracted extensive attention. Comparative genomics studies had found that the domestic dog genome exhibited adaptation to a starch-rich diet (Axelsson et al., 2013). However, adaptive changes in gut microbiota that co-evolve with the host have not received sufficient attention.

Previous studies of gut microbiota diversity found differences in the abundance bacterial genera in domestic dogs and wolves. Dogs feeding on human food leftovers and commercial pet foods hold indeed more amylolytic gut bacteria such as *Ruminococcaceae*, *Desulfuromonadaceae*, and *Faecalibacterium* (Lyu et al., 2018), while wolves feeding on raw carcasses possess more *Clostridium sensu stricto* 1 (Liu et al., 2021). By further comparing the intestinal microbial functions of domestic dogs and wolves, it was found that dietary habits promoted the differentiation of intestinal microbes of domestic dogs and wolves. More microbial groups related to carbohydrate metabolism were observed in domestic dogs (Lyu et al., 2018). These studies provided important data for understanding the adaptation of wolves and domestic dogs to different foods. However, due to the limitation of sample collection, most of the previous reports focused on captive wolves. The gut microbes of captive and wild wolves may differ due to their different living environments. Comparative studies on wild wolves, captive wolves and domestic dogs can help to understand the effects of captive environment on gut microbes of wolves, and to comprehensively evaluate the adaptive changes of gut microbes under domestication and food changes. Therefore, in this paper, comparisons of gut microbial metagenomes in wild wolves, captive wolves and domestic dogs were performed to look for adaptive changes in gut microbial community structure, microbial gene composition and functions related to food and environmental changes. This will provide scientific basis for the study of the synergistic evolution and symbiosis of mammals and their gut microbes.

## Materials and methods

### Sample collection

Fresh fecal samples of 3 wild wolves (gender was unknown, named CLW1N, CLW2N and CLW3N, classified into group CLW), 4 captive wolves (2 females and 2 males, named CLC1M, CLC2M, CLC3F and CLC4F, classified into group CLC) and 4 domestic dogs (2 females and 2 males, named CLF2M, CLF3M, CLF4F and CLF5F, classified into group CLF) were collected in winter in Hulun Lake Nature Reserve in Inner Mongolia, China (Supplementary Table 1). Wild wolves were tracked by using GPS collars in winter with snow on the ground. The wolves’ behavior was observed by using telescopes and drones during the tracking. Fresh feces of wild wolves were collected on the wolves’ footprint chains after they left. The fecal samples were further identified by sequencing and alignment analysis of mitochondrial cyt b gene and cox 1 gene. The local temperature at the time of sample collection was below −20°C, which ensured fecal samples freshness. Captive wolves were raised from pups rescued by grassland authorities in Hulun Lake Nature Reserve. The main food of captive wolves were dead sheep and live chickens. Sheep were killed and fed to wolves including skin and internal organs. The feeding time was not fixed. Generally, captive wolves would be feed again after they eat all the food. Dogs’ fecal samples were collected from native village dogs. They had individual owners and were free-range by herders on the grassland. Their food was mainly herders’ food residue, including pasta (wheat flour products and corn flour products), residual sheep bones, beef bones and chicken bones, and a small amount of vegetables, mainly potatoes and cabbage. They were fed once a day in the afternoon. Captive wolves and domestic dogs were healthy adults and samples were collected in the morning before feeding. They were not infected with any digestive diseases or treated with antibiotics or probiotics during the 3 months prior to sample collection. Samples were stored in sterilized plastic centrifuge tubes, sent to the laboratory on dry ice, and stored at-80°C until genomic DNA extraction. This study was conducted under the regulations of the Bioethics Committee of Qufu Normal University and complied with the regulations of the China Wildlife Conservation Association, Endangered Species Scientific Commission, P. R. China, and the relevant requirements of Chinese laws. No harm was done to the animals and their habitat during sample collection.

### Genomic DNA extraction, DNA library construction, and sequencing

Genomic DNA was extracted from fecal samples using the QIAamp DNA Fecal Genomic DNA Extraction Kit (Qiagen). The quality and concentration of DNA were quantified using NanoDrop 2000c. The purity and integrity of DNA were examined by agarose gel electrophoresis. The qualified DNA samples were randomly cut into fragments of approximately 350 bp in length by Covaris ultrasonic fragmentation, and the libraries were prepared by PCR amplification. Agilent 2100 was used to detect the INSERT size of the library. Qubit 3.0 and Q-PCR were used to accurately quantify the effective concentration of the library (library effective concentration >3 nM). After the library inspection, different libraries were pooling according to the effective concentration and target data amount and sequenced by using Illumina HiSeq 2500.

### Data processing and bioinformatics analysis

Reads with low quality base and high N-base ratio and with an adapter overlap exceeding the threshold were removed from raw data. SoapAligner software was used for alignment with the host database to filter out the reads that may come from the host and to filter for the clean data. Assembly was performed using SOAP denovo assembly software. For a single sample, K-mer = 55 was selected for assembly to obtain the assembly sequence (Scaffolds). Scaffolds were disrupted from the N junction to obtain N-free sequence fragments (Scaftigs). The clean data of each sample after quality control was aligned to the Scaftigs of each sample to filter out the PE reads that were not utilized. The unused PE reads of each sample were pooled together, and K-mer = 55 was selected for mixed assembly (NOVO_MIX). The N-free Scaftigs sequence was obtained by interrupting the mixed assembled Scaffolds from the N junction. For Scaftigs generated by single sample and hybrid assembly, fragments below 500 bp were filtered out for statistical analysis and subsequent gene prediction.

Open reading frames (ORFs) were predicted by using MetaGeneMark software from Scaftigs (≥500 bp), and ORFs less than 100 nt in length were filtered out. CD-HIT software was used to remove the redundant ORFs and to obtain the non-redundant initial gene catalogue. Clustering was performed with identity = 95% and coverage = 90%, and the longest sequence was selected as the representative sequence. SoapAligner was used to align the clean data of each sample to the initial gene catalogue, and the number of aligned reads in each sample were calculated. The genes whose supporting reads ≤2 in each sample were filtered out to obtain the gene catalogue (unigenes) for subsequent analysis. Based on the number of reads and the length of the alignment, the abundance information of each gene in each sample were calculated with the formula $G_{k}=\frac{r_{k}}{L_{k}}\times \frac{1}{\sum_{i=1}^{n}\frac{r_{i}}{L_{i}}}$, in which r is the reads number of the gene in the alignment, and L is the length of the gene. Venn diagram was used to show the number of common and unique genes among groups (CLW, CLC and CLF). Wilcoxon signed-rank tests were performed to investigate the difference in the number of annotated genes among groups.

### Species annotation and comparison between groups

The DIAMOND software was used to align unigenes to bacteria, fungi, archaea and viruses sequences selected from the NR database of NCBI (blastp, evalue ≤1e–5). The alignment results of evalues ≤ (minimum evalue × 10) were selected for species annotation. The LCA algorithm of MEGAN software took the taxonomic level before the first branch as the species annotation information of the sequence. Based on the LCA annotation results and gene abundances, the abundance information of each sample at each taxonomic level (phylum, class, order, family, genus and species) was obtained. The abundance of a species in a sample was the sum of the abundance of genes annotated to that species. Alpha diversity (Shannon index and Simpson index) test of each group was performed by using Qiime software. Beta diversity, estimated with Bray-Curtis metrics, was calculated using R (version 2.15.3) to compare the composition of microbial communities among different groups. The relative abundance of different groups at each taxonomic level were compared by Wilcoxon signed-rank test and MetaStat test. Anosim analysis, PCA and NMDS dimensionality reduction analysis, cluster analysis based on Bray-Curtis distance were performed to test for the similarity among the groups. Linear discriminant analysis effect size (LEfSe) analysis was performed to search for the representative biomarkers.

### Gene function annotation and comparison between groups

The Unigenes was aligned to the KEGG, eggNOG, and CAZy databases using the DIAMOND software (blastp, evalue 1e–5). The KEGG database was divided into 6 levels (level 1, level 2, level 3, ko, ec and module), the eggNOG database was divided into 3 levels (level 1, level 2, and og), and the CAZy database was divided into 3 levels (level 1, level 2 and ec). The comparison result with the highest score (one HSP >60 bits) was selected for subsequent analysis. The relative abundances of different functional levels were counted (the relative abundance of each functional level was the sum of the relative abundances of genes annotated to that functional level). The differences in functional abundance between the groups were compared by MetaStat test. The similarity of annotation function between groups was compared by PCA, NMDS and cluster analysis based on Bray-Curtis distance.

## Results

### Basic data statistics

A total of 58,820.98 Mbp clean data was obtained by Illumina HiSeq sequencing and preprocessing. Data effective was above 99.96%. By SOAP denovo assembly, a total length of 761.93Mbp and a total number of 453,461 Scaftigs were obtained for statistical analysis and subsequent gene prediction. By using MetaGeneMark for ORF prediction, a total of 418,264 ORFs were obtained and the total length of the ORFs (gene catalogue) was 264.15 Mbp (Supplementary Table 2).

### Comparison of gene abundance between groups

The abundance of annotated unigenes in group CLF (153,450.25 ± 14,789.94) was higher than that of group CLC (146,185.5 ± 16,823.42) and significantly higher than that of group CLW (135,373.3 ± 5,240.67; Wilcoxon signed-rank test, *p <* 0.05; Supplementary Figure 1). Venn diagram showed that the number of common genes among groups exceeded the number of unique genes in each group, which indicated that the common core gene group was dominant among wild wolves, captive wolves and domestic dogs. Among the three groups, the number of genes unique to CLF was the highest while the number of genes unique to CLW was the lowest. Groups CLC and CLF had the highest number of genes in common (Supplementary Figure 2).

### Species annotation and comparison among groups

The NR database annotation of unigenes showed that the proportion of fungi, archaea and viruses was less than 1%, the proportion of bacteria was 82–95%. A total of 74 bacterial phyla, 323 families, 983 genera, 3,832 species and a certain number of unclassified species were annotated in the intestinal microbiota of wolves and domestic dogs. At the phylum level, the proportions of unclassified species in groups CLW, CLC and CLF were 13.71, 13.37 and 9.66%, respectively. At the family level, the proportions of unclassified species in groups CLW, CLC and CLF were 24.22, 23.64 and 20.88%, respectively. At the genus level, the proportions of unclassified species in groups CLW, CLC and CLF were 27.31, 28.38 and 24.17%, respectively. Anosim analysis results based on each taxonomic level showed that the differences among groups CLW, CLC and CLF were higher than the differences within the group (*R* > 0, *p >* 0.05; Supplementary Figure 3). The Shannon index and Simpson index showed a pattern of CLF > CLC > CLW by alpha diversity test and comparison among groups (Supplementary Table 3).

**Figure 1:**
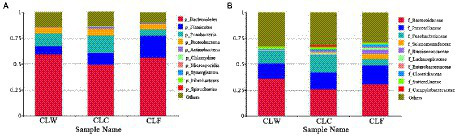
Relative abundance of annotated genes at phylum and family levels. The top 10 phylum **(A)**, and family **(B)**, with the highest abundance in each group were selected and the rest were set to others.

The phyla with the highest abundance were Bacteroidetes, Firmicutes, Fusobacteria, Proteobacteria and Actinobacteria. Bacteroidetes accounted for more than 40% in each sample while Firmicutes (7–37%) and Fusobacteria (1–25%) showed great differences in each sample (Figure 1A). Wilcoxon signed-rank tests found that the abundance of Firmicutes was higher in CLF (21.39%) than in CLC (11.20%) and significantly higher than that in CLW (7.82%; *p* = 0.027). The abundance of Fusobacteria in group CLC (16.49%) was higher than that in group CLW (12.14%) and was significantly higher than that in group CLF (6.09%; *p* = 0.04).

The highest abundant families were Bacteroidaceae, Prevotellaceae, Fusobacteriaceae, Selenomonadaceae, Ruminococcaceae (Figure 1B). Wilcoxon signed-rank tests showed that the abundance of Bacteroidaceae in group CLW was higher than that in groups CLC and CLF (*p >* 0.05). The abundance of Fusobacteriaceae in group CLC was higher than that in group CLW and significantly higher than that in group CLF (*p <* 0.05). The abundance of Ruminococcaceae in group CLF was significantly higher than that in groups CLC and CLW (*p <* 0.05).

*Bacteroides*, *Fusobacterium*, *Prevotella*, *Megamonas*, *Paraprevotella*, *Faecalibacterium*, *Clostridium*, *Campylobacter*, *Sutterella*, *Escherichia* were the genera with higher abundance (Supplementary Figure 4A). The abundance of *Bacteroides* in group CLW was higher than that in groups CLC and CLF (*p >* 0.05). The abundance of *Fusobacterium* in group CLC was higher than that in group CLW and significantly higher than that in group CLF (*p <* 0.05). The abundance of *Paraprevotella* in group CLW was significantly higher than that in groups CLC and CLF (*p <* 0.05). The abundance of *Faecalibacterium* in group CLF was significantly higher than that in groups CLC and CLW (*p <* 0.05).

**Figure 2:**
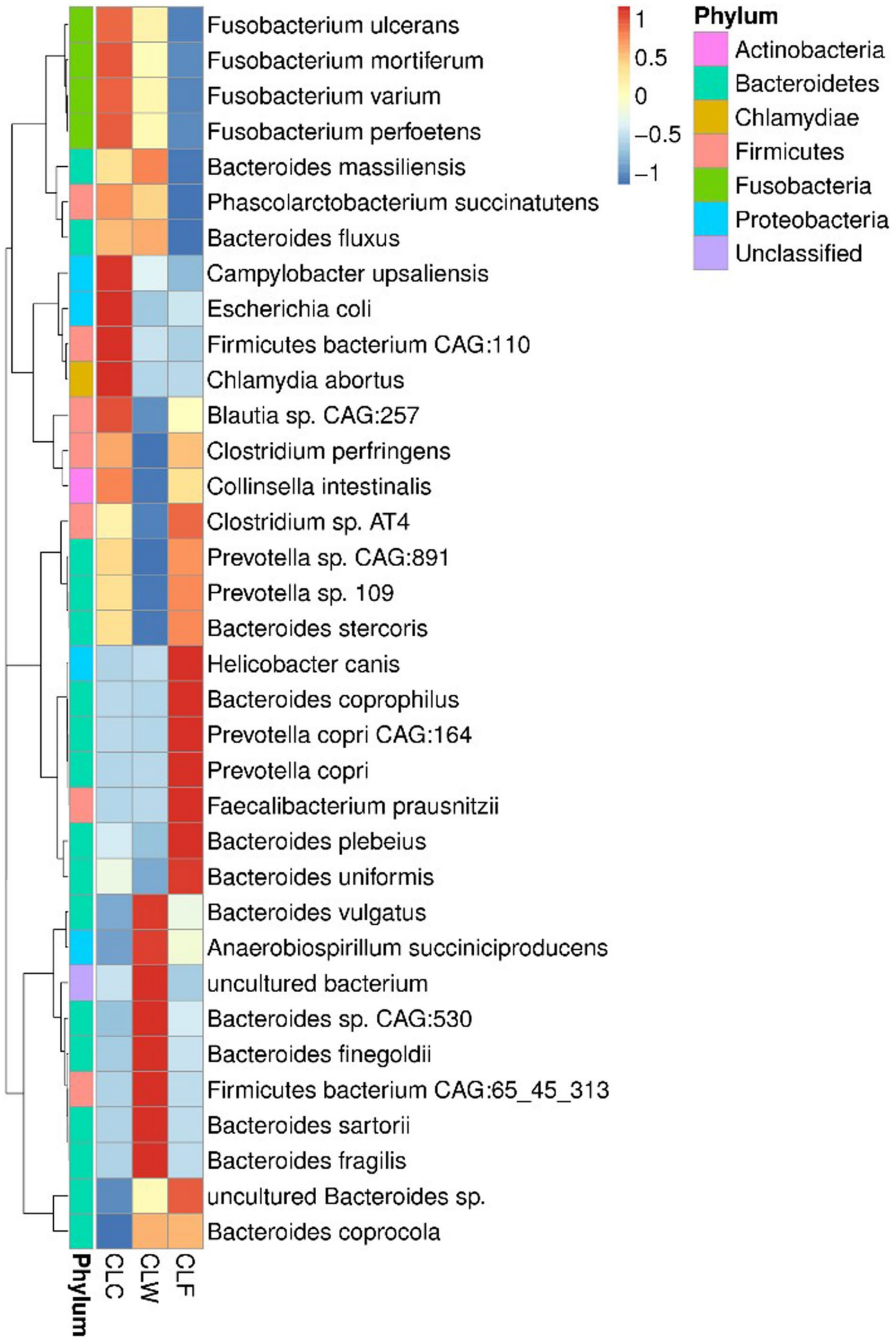
Heat map showed the difference in annotated gene abundance at species level among groups (showing the relative abundance of the top 35 species).

*Prevotella* sp. CAG:891 and *Fusobacterium perfoetens* were the most abundant bacteria in the gut of wolves and domestic dogs (Supplementary Figure 4B). Wilcoxon signed-rank tests showed that there was no significant difference in the abundance of *Prevotella* sp. CAG:891 among the three groups. Although there was also no significant difference in the abundance of *Fusobacterium perfoetens* among the three groups, the abundance of other bacteria with high abundance in genus *Fusobacterium* was significantly higher in the group CLC. For example, the abundance of *Fusobacterium ulcerans*, *Fusobacterium mortiferum*, and *Fusobacterium varium* in the group CLC were higher than that in the group CLW and significantly higher than that in the group CLF (*p <* 0.05). At the same time, the abundance of a variety of bacteria with high abundance in genera *Clostridium* and *Collinsella* were significantly lower in group CLW. For instance, the abundance of *Clostridium* sp. AT4 in group CLF was higher than that in group CLC and significantly higher than that in group CLW (*p <* 0.05). The abundance of the *Collinsella intestinalis* was higher in the group CLC than in the group CLF and significantly higher than that in the group CLW (*p <* 0.05). On the contrary, the overall abundance of genus *Bacteroides* in group CLW was higher than that in the other two groups. However, the relative abundance of several bacteria in the genus *Bacteroides* was not consistent among the three groups. For example, the relative abundance of *Bacteroides massiliensis* in group CLW was higher than that in group CLC and significantly higher than that in group CLF (*p <* 0.01). The relative abundance of *Bacteroides vulgatus* and *Bacteroides fragilis* in group CLW was significantly higher than that in group CLC and group CLF (*p <* 0.05). On the contrary, the relative abundances of *Bacteroides stercoris* and *Bacteroides uniformis* in groups CLF and CLC were significantly higher than those in group CLW (*p <* 0.05). In addition, the abundance of *Anaerobiospirillum succiniciproducens* with a higher abundance in group CLW was higher than that in the group CLF and significantly higher than that in the group CLC (*p <* 0.05). The abundance of *Faecalibacterium prausnitzii* in group CLF was significantly higher than that in groups CLC and CLW (*p <* 0.05). It was intersting that the abundance of *Escherichia coli* in groups CLC and CLF was higher than that in group CLW, although the difference was not significant (*p >* 0.05; Figure 2).

### PCA, NMDS and clustering analysis based on species abundance

**Figure 3:**
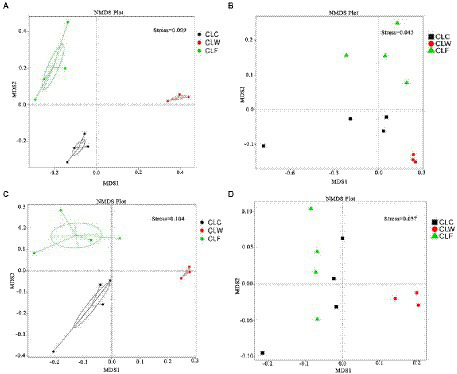
NMDS analysis based on species abundance **(A)**, function abundance annotated at the KEGG level ko **(B)**, function abundance annotated at eggNOG level og **(C)**, and function abundance annotated at CAZy level ec **(D)**.

**Figure 4:**
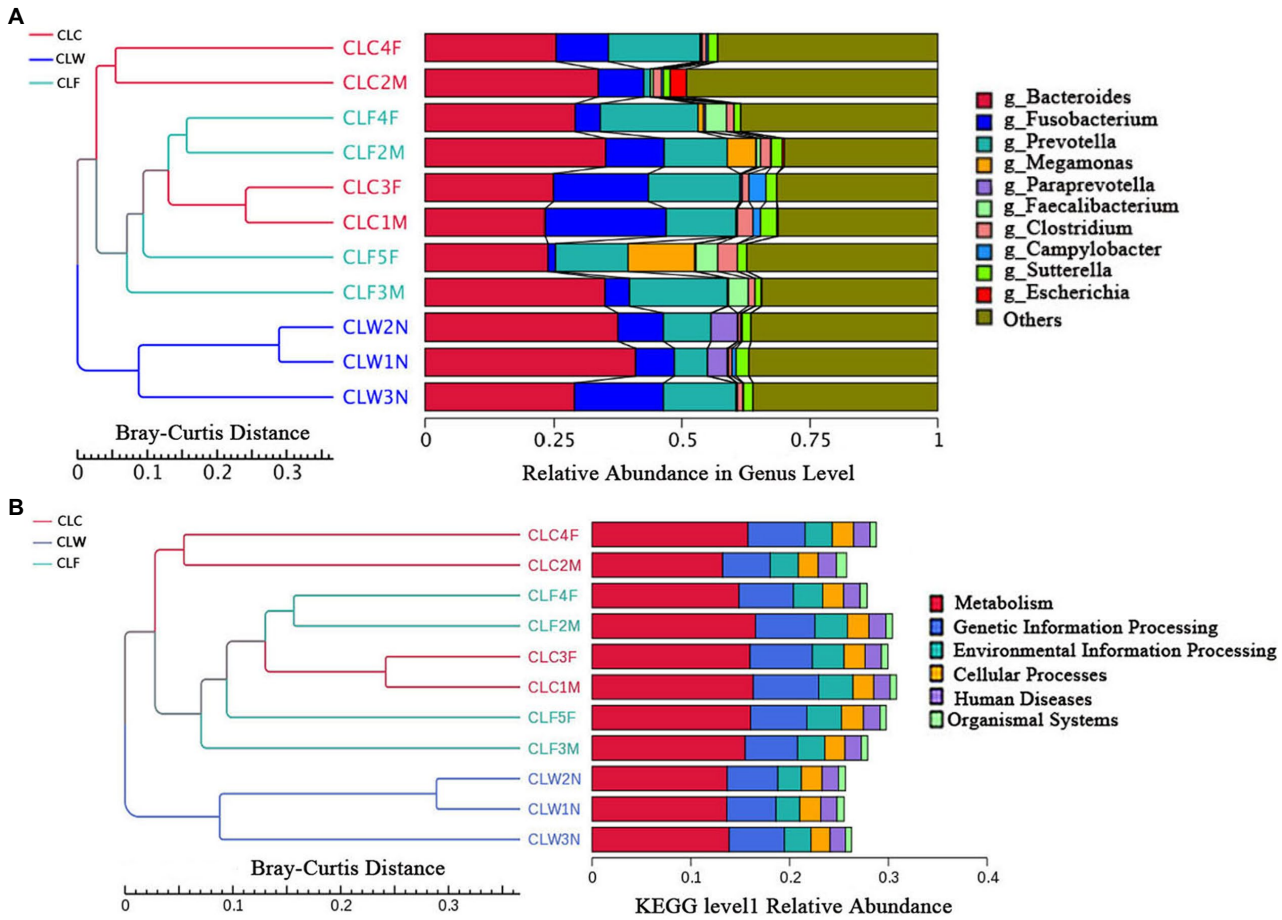
Clustering tree based on Bray-Curtis distance and annotated species abundance at genus level **(A)**, and annotation function abundance at KEGG level 1 **(B)**.

The results of PCA and NMDS analysis based on species abundance annotation at each level showed that samples from each group clustered independently (Figure 3A; Supplementary Figure 5). The results of cluster analysis based on Bray-Curtis distance showed that the samples from group CLW clustered into one branch and samples from groups CLC and CLF mixed into the other branch (Figure 4A; Supplementary Figure 6).

**Figure 5:**
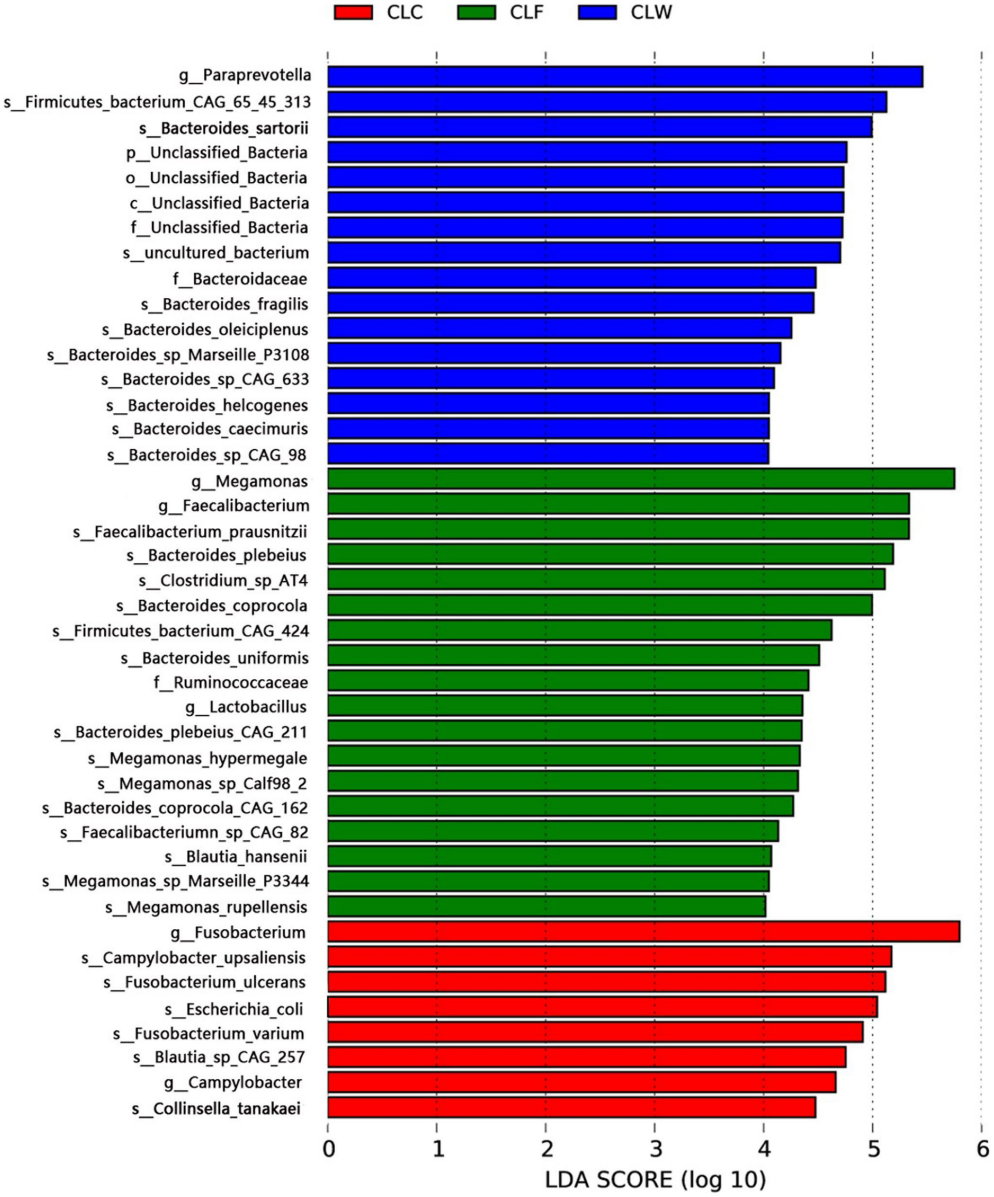
LEfSe analysis showed respective biomarkers in groups CLW, CLC and CLF.

The LDA branching diagram showed that the three groups had their own unique biomarkers. Biomarkers of group CLW were mainly belong to genus *Bacteroides* (family Bacteroidaceae, phylum Bacteroidetes), including *Bacteroides caecimuris*, *Bacteroides fragilis*, *Bacteroides helcogenes*, *Bacteroides oleiciplenus* and *Bacteroides sartorii*. Biomarkers of group CLF were mainly in phylum Firmicutes, included genus *Faecalibacterium* in the family Ruminococcaceae (including *Faecalibacterium prausnitzii* and *Faecalibacteriumn* sp. CAG82), genus *Megamonas* in the family Selenomonadaceae (including *Megamonas hypermegale* and *Megamonas rupellensis*), as well as *Blautia hansenii* in the family Lachnospiraceae and *Clostridium* sp. AT4 in the family Clostridiaceae. Biomarkers of group CLC were mainly the genus *Fusobacterium* in the family Fusobacteriaceae, phylum Fusobacteria, including *Fusobacterium ulcerans* and *Fusobacterium varium* (Figure 5).

### Gene function annotation

In the KEGG database, the intestinal microbial genes of wolves and domestic dogs were mainly enriched in three metabolic pathways: metabolism (13.47%), genetic information processing (4.53%) and environmental information processing (3.79%). Among them, carbohydrate metabolism, amino acid metabolism, nucleotide metabolism, metabolism of cofactors and vitamins and energy metabolism in metabolism pathway, membrane transport in environmental information processing pathway and translation in genetic information processing pathway enriched the highest number of genes (Supplementary Figure 7).

In the eggNOG database, a large number of genes were functionally unknown (28.37% of unigenes). Functional clusters with a higher number of enriched genes included Replication, recombination and repair, cell wall/membrane/envelope biogenesis, amino acid transport and metabolism, carbohydrate transport and metabolism, translation, ribosome structure and biogenesis, transcription, inorganic ion transport and metabolism and energy production and conversion (Supplementary Figure 8). In the CAZy database, genes were mainly enriched in Glycoside Hydrolases (GHs) and glycosyl transferases (Supplementary Figure 9).

### Comparison of annotation function abundance among groups

**Figure 6:**
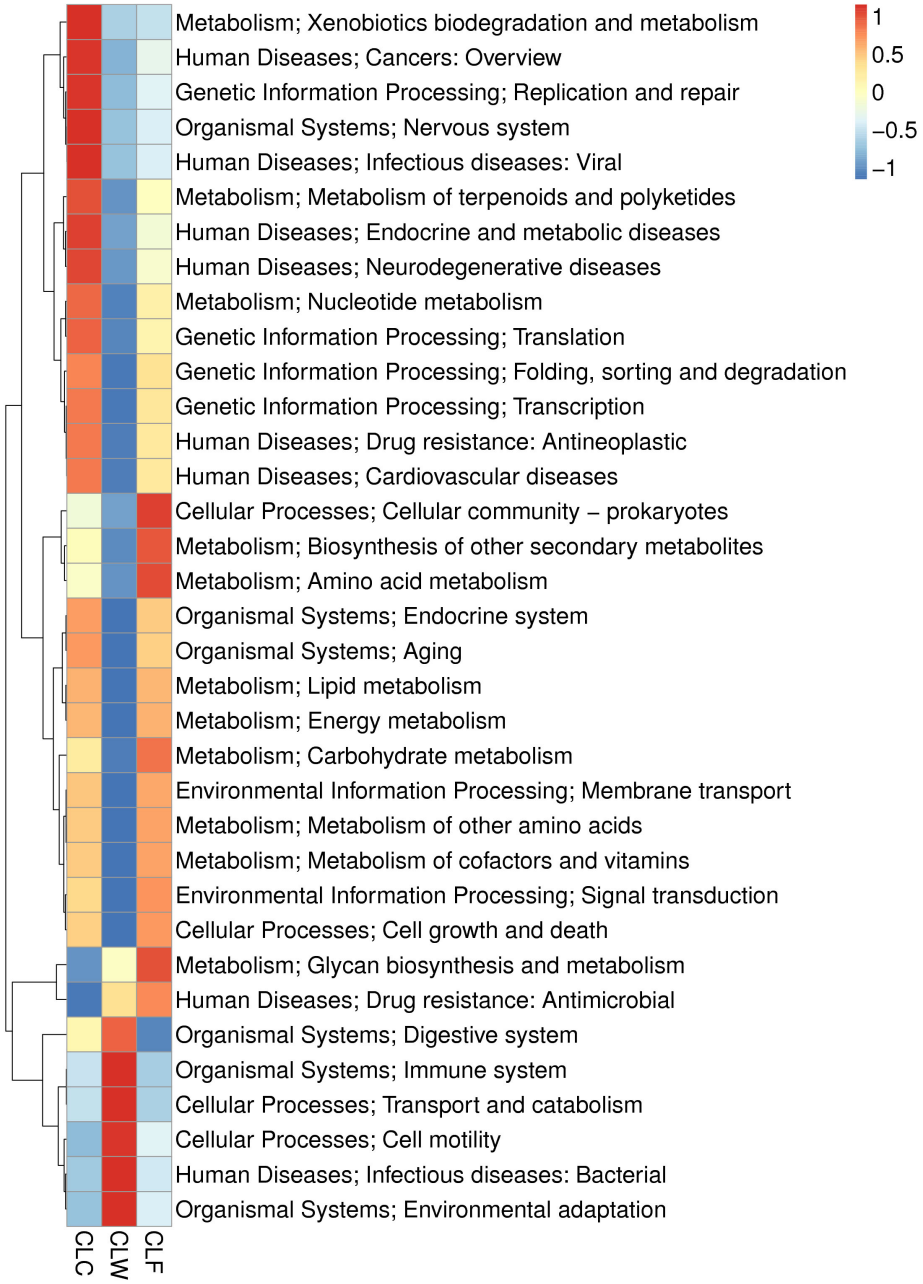
Heat map showed the difference in functional abundance of groups CLW, CLC and CLF annotated at KEGG level 2.

MetaStat test showed that in the annotation results of KEGG database (level 2), the abundance of carbohydrate metabolism, biosynthesis of other secondary metabolites, metabolism of other amino acids in metabolism, signal transmission in environmental information processing, transcription in genetic information processing, cardiovascular diseases, drug resistance: antimicrobial and neurodegenerative diseases in human diseases, and aging and endocrine system pathways in organic systems were significantly higher in CLC group than in CLW group (*q* < 0.05); the abundance of environmental adaptation in organic systems and infectious diseases: bacterial pathway in human diseases was significantly higher in group CLW than in group CLC (*q* < 0.05); the abundance of carbohydrate metabolism, lipid metabolism, amino acid metabolism, biosynthesis of other secondary and metabolism of other amino acids in metabolism, signal transduction in environmental information processing, cell growth and death in cellular processes, endocrine and metabolic diseases in human diseases, and endocrine system in organismal systems were significantly higher in group CLF than in group CLW (*q* < 0.05; Figure 6). Interestingly, most of the functions with higher annotated abundance did not show significantly different between groups CLC and CLF (Supplementary Table 4).

KEGG pathway annotation showed that compared with the group CLW, a large number of unique metabolic pathways and enzymes were annotated in the group CLF. For example, in the biosynthesis of amino acids pathway, S-sulfo-l-cysteine synthase (EC:2.5.1.144) was unique to the CLF group, which was mainly involved in the metabolism of cysteine and methionine and amino acid biosynthesis. In the carbon metabolism pathway, malate synthase (EC:2.3.3.9), glutamate--glyoxylate aminotransferase (EC:2.6.1.4 2.6.1.2 2.6.1.44), isocitrate lyase (EC:4.1.3.1), acetoacetyl-Coa Reductase (EC:1.1.1.36) and some other enzymes were only annotated in group CLF, but 3-hydroxyButyryl-Coa dehydratase (EC:4.2.1.55) was only annotated in the group CLW (Supplementary Figure 10). In the starch and sucrose metabolism pathway, hexokinase (EC:2.7.1.1) and alpha-trehalase (EC:3.2.1.28) were detected only in the group CLF while glycogen synthase (EC:2.4.1.11) was only detected in the group CLW (Supplementary Figure 11). In the glycolysis/gluconeogenesis pathway, glucose-6-phosphate 1-epimerase (EC:5.1.3.15) and phosphate dehydrogenase (EC:1.2.1.-) were only found in the group CLF (Supplementary Figure 12). Interestingly, in the fat acid metabolism pathway, 3-hydroxyacyl-CoA dehydrogenase (EC:1.1.1.35), enoyl COA hydratase (EC:4.2.1.17), acetyl CoA acyltransferase (EC:2.3.1.16) and some other enzymes were also only found in the CLF group. These enzymes were mainly concentrated in the fat acid degradation pathway. Similarly, by comparing the pathway annotation results between groups CLC and CLW, it was also found that some enzymes in biosynthesis of amino acids, carbon metabolism, fatty acid metabolism and other pathways were only annotated in CLC group (Supplementary Figure 13).

**Figure 7:**
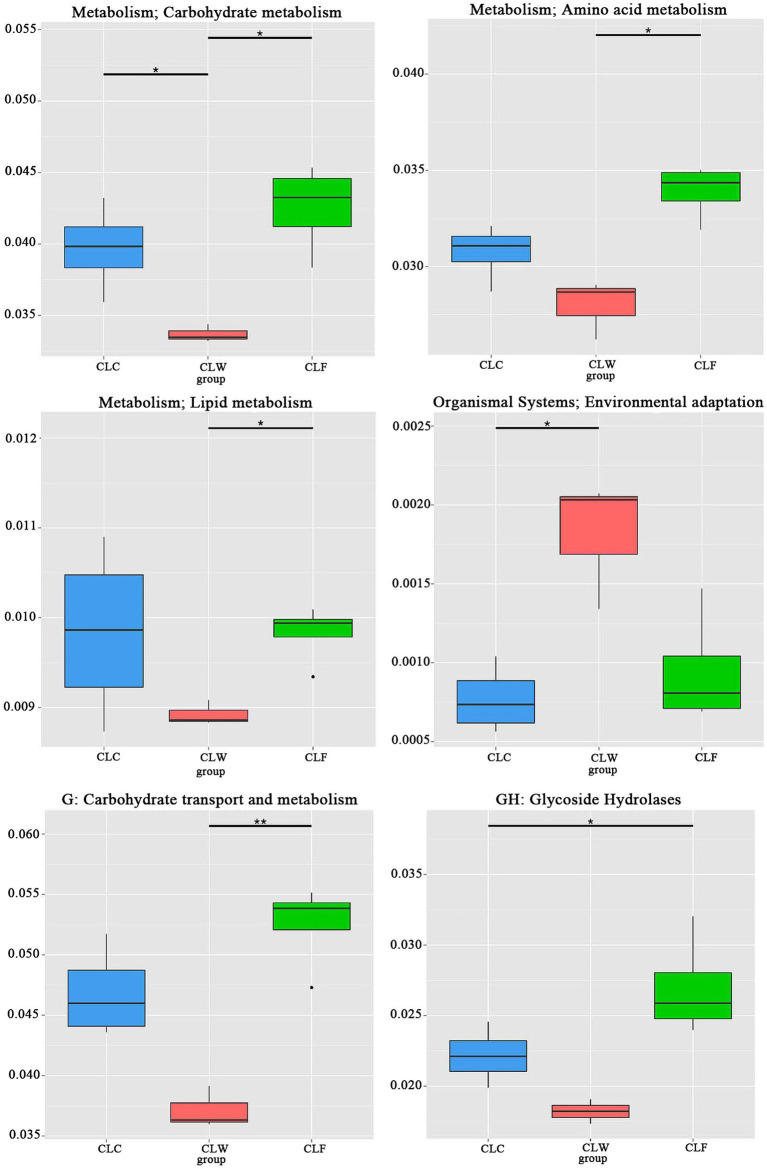
Metastat test revealed significantly difference in function abundance among groups CLW, CLC and CLF annotated at eggnog level 1. “*” represented significant difference and “**” represented extremely significant difference.

Among the functions annotated in the eggNOG database (level 1), the annotation results of groups CLW and CLF were significantly different in the pathways of carbon transport and metabolism, amino acid transport and metabolism, nucleoside transport and metabolism, inorganic ion transport and metabolism, signal transmission mechanisms and transcription, and posttranslational modification (CLF > CLW, MetaStat test *q* < 0.05), but no significant difference was detected between groups CLC and CLF and between groups CLC and CLW (Figure 7). Annotation results in both KEGG and eggNOG functional databases showed that the abundance of genes annotated as unknown function in group CLW (74.18% of unigenes in KEGG level 1, 26.67% of unigenes in eggNOG level 1) was significantly higher than that in group CLF (MetaStat test *q* < 0.05).

**Figure 8:**
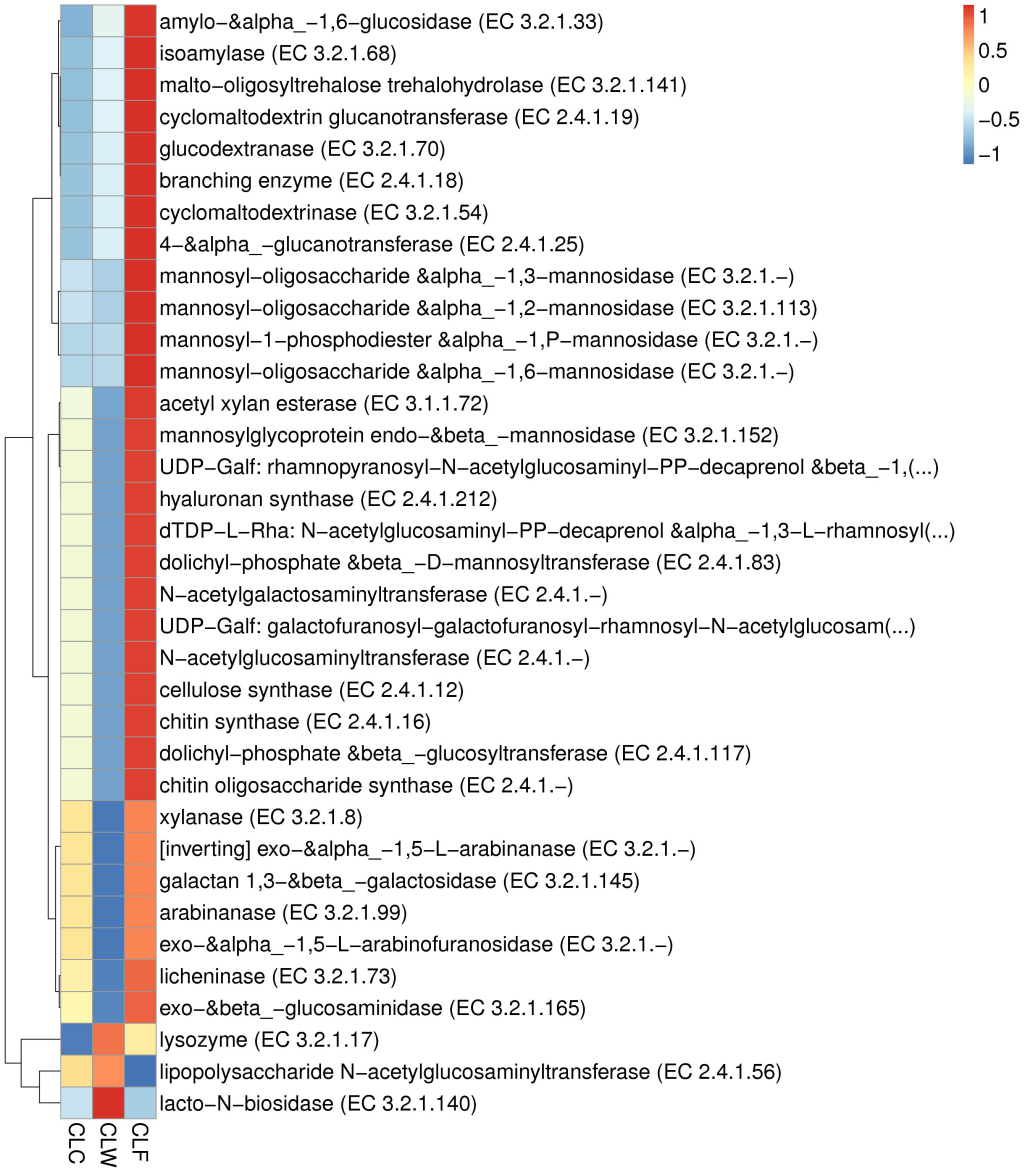
Heat map showed the difference in functional abundance of groups CLW, CLC and CLF annotated at CAZy level ec.

In the CAZy database (level ec), the abundance of 244 enzymes were significantly different between the groups CLF and CLW, and 177 of these enzymes had a significantly higher abundance in the group CLF than in the group CLW (MetaStat test *q* < 0.05). Sixty enzymes were extremely significantly different between groups CLF and CLW (MetaStat test *q* < 0.01). The abundances of 49 enzymes in the group CLF, such as alpha-L-arabinofuranosidase (EC 3.2.1.55), beta-xylosidase (EC 3.2.1.37), alpha-glucosidase (EC 3.2.1.20), alpha-1,2-L-arabinofuranosidase (EC 3.2.1.-), beta-1,3-xylosidase (EC 3.2.1.-), beta-glucosidase (EC 3.2.1.21), beta-primeverosidase (EC 3.2.1.149), beta-galactosidase (EC 3.2.1.23), endoglucanase (EC 3.2.1.4), were extremely significantly higher than that of the group CLW (MetaStat test *q* < 0.01). Significant differences in the abundance of 140 enzymes were detected between groups CLC and CLW, and 108 of these enzymes had a significantly higher abundance in the group CLC than in the group CLW (MetaStat test *q* < 0.05). Only 27 enzymes were significantly different between groups CLF and CLC (CLF > CLC, *q* < 0.05; Figure 8; Supplementary Table 5).

### PCA, NMDS and clustering analysis based on function abundance

PCA and NMDS analysis based on the functional abundance annotated in KEGG, eggNOG and CAZy databases showed that samples from the group CLW was separated from samples from the other two groups. PCA and NMDS analysis based on the KEGG and eggNOG annotations showed that the samples from the groups CLC and CLF were clustered separately, while analysis based on the CAZy annotations showed that the samples from groups CLC and CLF were mixed together (Figures 3B–D; Supplementary Figure 14).

Clustering analysis based on Bray-Curtis distance matrix and KEGG, eggNOG and CAZy annotations showed that samples from the group CLW was clustered into one branch and samples from groups CLA and CLF were clustered into the other branch. This result was the same as the analysis at the species level (Figure 4B; Supplementary Figure 15).

## Discussion

Through species annotation, it is showed that wild wolves, captive wolves and domestic dogs share more core microbiota than their respective endemic microbiota, which indicates that the gut core microbiota is effectively preserved despite the differences in food. *Bacteroides*, *Fusobacterium*, *Prevotella*, *Megamonas* and *Paraprevotella* are the genera with the highest abundance of intestinal microbiota in wolves and dogs, which is consistent with previous studies (Wu et al., 2017; Lyu et al., 2018; Liu et al., 2021). The difference analysis among groups showed that the abundances of *Fusobacterium*, *Faecalibacterium* and *Paraprevotella* were significantly different among the three groups. LESfe test showed that *Bacteroides* (mainly *Bacteroides fragilis* and *Bacteroides vulgatus*) and *Fusobacterium* were biomarkers of group CLW and group CLC, respectively, while the biomarkers of group CLF were mainly *Faecalibacterium* and *Megamonas*. *Bacteroides* maintain a complex and generally beneficial relationship with the host when retained in the gut, but when they escape this environment they can cause pathology in humans (Wexler, 2007). *Bacteroides fragilis* is mainly parasitizes on the mucosal surface and its number of live cells is generally 10–100 times less than that of other species of the genus *Bacteroides* in the gut (Salyers, 1984). Genomic and proteomic analysis revealed that *Bacteroides fragilis* can sense and adapt to complex systems of nutrient availability, excrete toxic substances through multiple pump systems and can control the reproduction of other pathogens by affecting host immunity (Kuwahara et al., 2004; Wexler, 2007). The high abundance of *Bacteroides fragilis* in the intestinal of wild wolves may be related to the intestinal immune function of wild wolves. *Bacteroides vulgatus*, another bacterium in genus *Bacteroides*, is significantly associated with intestinal lipid metabolism (Yoshida et al., 2018). The higher abundance of *Bacteroides vulgatus* in the group CLW may be related to the high-protein and high-fat food composition of wild wolves. A variety of bacteria in genus *Bacteroides* are related to the metabolism of carbohydrate and polysaccharide (Hyun et al., 2012; Grondin et al., 2022). While observing higher abundance of some species of genus *Bacteroides* in the intestinal microbiota of wild wolves, higher abundance in some other species of genus *Bacteroides* in the domestic dog and captive wolves were also observed.

Genus *Faecalibacterium* is the representative biomarker for group CLF. Its abundance in group CLF is significantly higher than that in groups CLC and CLW (*p <* 0.05). Lyu et al. (2018) found that the abundance of *Faecalibacterium* associated to glucose fermentation was significantly increased in the feces of domestic dogs, which may be related to the complex polysaccharide diet of domestic dogs (Lopezsiles et al., 2012; Lyu et al., 2018). *Fusobacterium varium* is one of the main anaerobic bacteria producing butyric acid in the gut. It plays an important role in glucose metabolism (Potrykus et al., 2007). The methylate, the hydroxyglutarate and the aminobutyrate pathways involved by *Fusobacterium varium* are important pathways of intestinal amino acid catabolism (Buckel, 2001; Potrykus et al., 2008; Neish, 2009; Ramezani et al., 2011). As dogs in the bones and raw food diets (BARF) group were fed a significantly higher amount of protein and fat, LefSe analysis showed a higher abundance of *Fusobacterium* in the dogs with a BARF diets compared to conventionally fed dogs (Schmidt et al., 2018; Xu et al., 2021). In the present study, the high abundance of *Fusobacterium* in captive wolves may be related to the production of butyrate and the regulation of amino acid and glucose metabolism.

Previous studies found that because the canine diet contained more complex polysaccharides than the wolf’s diet, the abundance of functional genes involved in valine, leucine and isoleucine biosynthesis and nitrogen metabolism in the canine gut microbiota and the abundance of genes encoding glycosyltransferase family 34, rum-binding Module family 25 and GH family 13 were significantly higher than those of captive wolves. At the same time, the abundances of six glycosyltransferases and five GHs which mainly act on the metabolic pathways of starch and sucrose in domestic dogs were also significantly higher than those in captive wolves (Lyu et al., 2018). In this study, we confirmed that the functional genes in the intestinal microbiome of domestic dogs were significantly enriched in the carbohydrate metabolism pathway of KEGG database (CLF > CLW, MetaStat test *q* < 0.05). In addition, the annotated genes in group CLF were also significantly enriched in the amino acid metabolism, glycan biosynthesis and metabolism pathways. In contrast, more genes annotated by gut microbes of wild wolves were enriched in immune system, environmental adaptation and other pathways. This reflected the adaption of gut microbes to living environment. Wild wolves likely have periods of starvation, encounter a higher pathogen load, and have significant more exercise compared to the wild counterparts, all of which can influence the microbiome. Annotation results in the professional carbohydrate metabolism database (CAZy database) further revealed the differences in carbohydrate metabolism among gut microbiota of wild wolves, captive wolves and domestic dogs. In the level ec of CAZy database, 177 enzymes were detected with significantly higher abundance in group CLF than that in group CLW (*q* < 0.05) and 48 enzymes of them showed extremely significant differences between groups CLF and CLW (CLF > CLW, *q* < 0.01). This fully indicated that due to the increase of starch compounds in food, the intestinal microbiota of domestic dogs showed significant adaptation to the increase of carbohydrate. Interestingly, significant differences in the abundance of 140 enzymes were detected between the groups CLC and CLW (*q* < 0.05). However, there were few differences between the groups CLC and the CLF. This indicated that the intestinal microbiome of wolves in captivity may have changed significantly from that in the wild due to the differences in the living environment and food.

Several previous studies explored the differences of gut microbiota between wild and captive mammals (Guan et al., 2017; Li et al., 2017; Wasimuddin et al., 2017; Chi et al., 2019; Gao et al., 2019; Guo et al., 2019) and discussed the adaptive changes of intestinal microbiota of wild animals after captivity. Other studies found that the impact of living environment on intestinal microbiota of wild mammals may exceed that of genetic relationships (Chen et al., 2022). In this study, we found that samples from the wild wolves, captive wolves and domestic dogs diverged from each other based on the PCA and NMDS analysis of the composition and function of intestinal microbiota. However, no matter based on the species composition of intestinal microbiota or based on the functional composition of intestinal microbiomes, clustering analysis all found that captive wolves and domestic dogs clustered into one branch, while wild wolves clustered into the other branch. This result suggested that wolves living in captivity were closer to domestic dogs in both species composition and functional composition of intestinal microbiota. This also indicated that the correlation between gut microbes and food and living environment may be significantly higher than that with genetic relationship. The fecal samples of group CLC were collected from captive wolves living in the same grassland as group CLW, however, the results found that the gut microbial composition and function of captive wolves were closer to that of domestic dogs. This result indicates that the gut microbiome of the captive wolves underwent adaptive changes in captivity due to the food and living environment. These changes may relate to various factors, such as captive wolves’ food sources that are mainly poultry, domestic livestock, with small activity space and strong anthropogenic interference. The future study will introduce more wild and captive wolves from other habitats to further explore the impact of captivity on wolves’ physiology and health.

The ability of wild animals to survive on food that may be infected by pathogens, and to show immunity to various diseases, is inseparable from their complex gut microbial community (Levin et al., 2021). By species annotation and functional annotation, we found a certain number of unclassified species and a certain number of genes that could not be annotated into existing metabolic pathways in the feces samples of wild wolves. This indicated that wildlife as a potential microbial resource pool needed to be further exploited by next-generation sequencing technology.

Sample collection is difficult due to the small population, large habitat and covert activity of wild carnivores. Therefore, the small sample size is a major limitation of this study. In order to minimizing environmental differences on gut microbes, the samples of captive wolves living on the same grassland as wild wolves were collected. To reduce differences in gut microbes due to sample numbers, sample numbers for captive wolves and domestic dogs were designed with reference to the number of wild wolves. The small sample number and the lack of a standardized diet of animals have limited the further in-depth analysis of the data obtained here. However, as the first attempt to compare the gut microbial species and functional diversity of wild wolves, captive wolves and domestic dogs, this study provide a scientific reference for future studies to explore the ecological adaptive mechanism in the captive and domestic process of wild predators.

## Data availability statement

The datasets presented in this study can be found in online repositories. The names of the repository/repositories and accession number(s) can be found at: https://www.ncbi.nlm.nih.gov/, PRJNA871950.

## Ethics statement

The animal study was reviewed and approved by Bioethics Committee of Qufu Normal University.

## Author contributions

LC: conceived, designed, performed the experiments and collected samples. LC and MS: wrote the paper. MS, DX, ZG, YS, SW, and YZ: analyzed the data and modify the manuscript. All authors contributed to the article and approved the submitted version.

## Funding

This study is supported by the National Natural Science Foundation of China (31400473).

## Conflict of interest

The authors declare that the research was conducted in the absence of any commercial or financial relationships that could be construed as a potential conflict of interest.

## Publisher’s note

All claims expressed in this article are solely those of the authors and do not necessarily represent those of their affiliated organizations, or those of the publisher, the editors and the reviewers. Any product that may be evaluated in this article, or claim that may be made by its manufacturer, is not guaranteed or endorsed by the publisher.

## Supplementary material

The Supplementary material for thsis article can be found online at: https://www.frontiersin.org/articles/10.3389/fmicb.2022.1027188/full#supplementary-material
