# Supplementary material for: Gut microbiome of captive wolves is more similar to domestic dogs than wild wolves indicated by metagenomics study
Source: Front Microbiol. 2022 Nov 1;13:1027188. doi: 10.3389/fmicb.2022.1027188 (PMC9663663; doi:10.3389/fmicb.2022.1027188)
Supplement: Supplementary file 1 [file Table_1.DOCX]

Supplementary Table 1 Samples information of the animals in this study

| Animal | Sex | Abbreviation | Group |
| --- | --- | --- | --- |
| wild wolf | unkonwn | CLW1N、CLW2N、CLW3N | CLW |
| captive wolf | male  female | CLC1M、CLC2M  CLC3F、CLC4F | CLC |
| domestic dog | male  female | CLF2M、CLF3M  CLF4F、CLF5F | CLF |
