# Supplementary material for: Gut microbiome of captive wolves is more similar to domestic dogs than wild wolves indicated by metagenomics study
Source: Front Microbiol. 2022 Nov 1;13:1027188. doi: 10.3389/fmicb.2022.1027188 (PMC9663663; doi:10.3389/fmicb.2022.1027188)
Supplement: Supplementary file 2 [file Table_2.DOCX]

Supplementary Table 2 Sequencing data, Scaftigs and ORFs information for each sample

| sample | Raw  Data | Clean Data | Scaftigs | | | ORFs | | |
| --- | --- | --- | --- | --- | --- | --- | --- | --- |
|  |  |  | Number | Total length  (Mbp) | Average length  (bp) | Number | Total length  (Mbp) | Average length  (bp) |
| CLC1M | 5281.35 | 5273.42 | 38314 | 60.05 | 1567.37 | 78099 | 53.9 | 690.2 |
| CLC2M | 5395.74 | 5379.07 | 93660 | 86.08 | 919.05 | 95244 | 47.98 | 503.78 |
| CLC3F | 5595.36 | 5591.79 | 35251 | 57.67 | 1635.88 | 72668 | 51.26 | 705.34 |
| CLC4F | 5578.05 | 5573.43 | 42617 | 79.37 | 1862.48 | 95450 | 71.03 | 744.19 |
| CLF2M | 5418.96 | 5415.18 | 21625 | 54.76 | 2532.31 | 61080 | 48.27 | 790.33 |
| CLF3M | 5030.83 | 5026.97 | 56420 | 96.06 | 1702.54 | 119618 | 86.1 | 719.82 |
| CLF4F | 5120.28 | 5116.46 | 40624 | 71.69 | 1764.61 | 87462 | 63.13 | 721.75 |
| CLF5F | 5305.56 | 5301.74 | 43609 | 78.77 | 1806.37 | 98259 | 70.23 | 714.71 |
| CLW1N | 5550.56 | 5545.77 | 29331 | 64.33 | 2193.33 | 73684 | 56.55 | 767.47 |
| CLW2N | 5129.12 | 5125.71 | 24587 | 52.7 | 2143.59 | 60242 | 46 | 763.65 |
| CLW3N | 5475.91 | 5471.44 | 25326 | 59.13 | 2334.67 | 65970 | 51.7 | 783.62 |
| MIX |  |  | 9286 | 8.32 | 895.62 | 13912 | 7.28 | 523.26 |
