# Supplementary material for: Gut microbiome of captive wolves is more similar to domestic dogs than wild wolves indicated by metagenomics study
Source: Front Microbiol. 2022 Nov 1;13:1027188. doi: 10.3389/fmicb.2022.1027188 (PMC9663663; doi:10.3389/fmicb.2022.1027188)
Supplement: Supplementary file 3 [file Table_3.DOCX]

Supplementary Table 3 Alpha diversity index calculated based on relative abundances of each group（Mean ± SD）

|  | CLF | CLC | CLW |
| --- | --- | --- | --- |
| Shannon index | 3.037±0.26 | 2.949±0.08 | 2.740±0.01 |
| Simpson index | 0.762±0.05 | 0.756±0.03 | 0.699±0.04 |
