# Supplementary material for: Gut microbiome of captive wolves is more similar to domestic dogs than wild wolves indicated by metagenomics study
Source: Front Microbiol. 2022 Nov 1;13:1027188. doi: 10.3389/fmicb.2022.1027188 (PMC9663663; doi:10.3389/fmicb.2022.1027188)
Supplement: Supplementary file 4 [file Table_4.DOCX]

Table S4 MetaStat tests between groups based on the function abundance annotated in the KEGG level 2, “ > ” and “ < ” indicates the difference of the abundance between groups; “*” indicates significant difference (q value < 0.05); “*” indicates significant difference (q value < 0.05)

| Name | CLW vs CLF | CLC vs CLF | CLC vs CLW |
| --- | --- | --- | --- |
| Human Diseases; Infectious diseases: Bacterial | - | - | CLC < CLW * |
| Metabolism; Amino acid metabolism | CLW < CLF * | - | - |
| Human Diseases; Endocrine and metabolic diseases | CLW < CLF * | - | - |
| Human Diseases; Neurodegenerative diseases | - | - | CLC > CLW * |
| Metabolism; Lipid metabolism | CLW < CLF * | - | - |
| Metabolism; Energy metabolism | CLW < CLF * | - | - |
| Cellular Processes; Cell growth and death | CLW < CLF * | - | - |
| Human Diseases; Drug resistance: Antineoplastic | - | - | CLC > CLW * |
| Organismal Systems; Endocrine system | CLW < CLF * | - | CLC > CLW * |
| Metabolism; Biosynthesis of other secondary metabolites | CLW < CLF * | - | CLC > CLW * |
| Genetic Information Processing; Transcription | - | - | CLC > CLW * |
| Organismal Systems; Environmental adaptation | - | - | CLC < CLW * |
| Metabolism; Carbohydrate metabolism | CLW < CLF * | - | CLC > CLW * |
| Environmental Information Processing; Signal transduction | CLW < CLF * | - | CLC > CLW * |
| Human Diseases; Cardiovascular diseases | - | - | CLC > CLW * |
| Metabolism; Metabolism of other amino acids | CLW < CLF * | - | CLC > CLW * |
| Organismal Systems; Aging | - | - | CLC > CLW * |
| Others | CLW > CLF * | - | CLC < CLW * |
