# Supplementary material for: Gut microbiome of captive wolves is more similar to domestic dogs than wild wolves indicated by metagenomics study
Source: Front Microbiol. 2022 Nov 1;13:1027188. doi: 10.3389/fmicb.2022.1027188 (PMC9663663; doi:10.3389/fmicb.2022.1027188)
Supplement: Supplementary file 5 [file Table_5.DOCX]

Table S5 MetaStat tests among groups based on the function abundance annotated in the CAZy level ec, “ > ” and “ < ” indicates the difference of the abundance between groups; “*” indicates significant difference (q value < 0.05); “**” indicates extremely significant difference (q value < 0.01)

| Name | CLF vs CLW | CLC vs CLW | CLF vs CLC |
| --- | --- | --- | --- |
| rhamnogalacturonan alpha-1,2-galacturono(...)_476 | - | CLC > CLW * | - |
| UDP-Glc: (glucosyl)lipopolysaccharide al(...)_195 | CLF > CLW * | CLC > CLW * | - |
| exo-beta-glucosaminidase (EC 3.2.1.165) | CLF > CLW * | - | - |
| lactose synthase (EC 2.4.1.22) | CLF < CLW ** | - | - |
| UDP-Gal: beta-galactoside alpha-1,4-gala(...)_180 | CLF < CLW * | - | - |
| alpha-galactosidase (EC 3.2.1.22) | CLF > CLW * | CLC > CLW * | - |
| 2-keto-3-deoxynononic acid hydrolase (EC 3.2.1.-) | CLF < CLW ** | - | - |
| beta-N-acetylhexosaminidase (EC 3.2.1.52) | - | CLC > CLW * | - |
| protein O-beta-xylosyltransferase (EC 2.4.2.26) | CLF > CLW * | - | - |
| UDP-Glc: 4-hydroxybenzoate 4-O-beta-gluc(...)_196 | CLF < CLW * | - | - |
| pullulanase (EC 3.2.1.41) | CLF > CLW * | - | CLF > CLC * |
| thiopeptidoglycan lyase (EC 4.2.2.-) | CLF > CLW * | CLC > CLW * | - |
| UDP-GlcA: baicalein 7-O-beta-glucuronosy(...)_200 | CLF < CLW * | - | - |
| stachyose synthase (EC 2.4.1.67) | CLF > CLW ** | - | - |
| UDP-glucuronosyltransferase (EC 2.4.1.17) | CLF < CLW * | - | - |
| alpha-1,3_1,4-L-fucosidase (EC 3.2.1.111) | CLF < CLW * | CLC < CLW * | - |
| sucrose:sucrose 1-fructosyltransferase (EC 2.4.1.99) | CLF > CLW * | - | - |
| 6-phospho-beta-galactosidase (EC 3.2.1.85) | CLF > CLW ** | CLC > CLW * | - |
| GDP-Man: Man2GlcNAc2-PP-dolichol alpha-1(...)_148 | CLF < CLW * | CLC < CLW * | - |
| dTDP-beta-2-deoxy-L-fucose: alpha-L-2-de(...)_279 | CLF < CLW * | - | - |
| exo-beta-1,4-glucanase _ cellodextrinase (EC 3.2.1.74) | CLF > CLW * | - | - |
| alpha-1,4-N-acetylgalactosaminyltransferase (EC 2.4.1.-) | CLF < CLW * | - | - |
| alpha-1,2-L-arabinofuranosidase (EC 3.2.1.-) | CLF > CLW ** | CLC > CLW * | - |
| beta-porphyranase (EC 3.2.1.178) | CLF > CLW * | - | - |
| cellobiohydrolase (EC 3.2.1.91) | - | CLC > CLW * | - |
| diglucosyl diacylglycerol synthase (EC 2.4.1.208) | CLF < CLW * | CLC < CLW * | - |
| strictosidine beta-glucosidase (EC 3.2.1.105) | CLF > CLW ** | CLC > CLW * | - |
| UDP-Xyl alpha-xylosyltransferase (EC 2.4.2.-) | CLF < CLW * | CLC < CLW * | - |
| Others | CLF < CLW ** | CLC < CLW * | - |
| pectin methylesterase (EC 3.1.1.11) | CLF > CLW * | CLC > CLW * | - |
| polygalacturonase (EC 3.2.1.15) | - | CLC > CLW * | - |
| hesperidin 6-O-alpha-L-rhamnosyl-beta-gl(...)_377 | CLF > CLW * | - | - |
| beta-1,6-glucanase (EC 3.2.1.75) | CLF > CLW * | - | - |
| maltotetraose-forming alpha-amylase (EC 3.2.1.60) | CLF > CLW * | - | CLF > CLC * |
| N-acetyllactosaminide beta-1,6-N-acetylg(...)_168 | CLF > CLW * | - | - |
| anhydrosialidase (EC 4.2.2.15) | CLF < CLW ** | - | - |
| alpha-1,2-L-fucosyltransferase (EC 2.4.1.69) | CLF > CLW * | CLC > CLW * | - |
| beta-agarase (EC 3.2.1.81) | CLF > CLW * | - | - |
| sialidase or neuraminidase (EC 3.2.1.18) | CLF < CLW ** | - | - |
| cyclomaltodextrinase (EC 3.2.1.54) | CLF > CLW * | - | CLF > CLC * |
| cycloinulo-oligosaccharide fructanotransferase (EC 2.4.1.-) | CLF > CLW * | - | - |
| undecaprenyl-diphosphooligosaccharide芒?...)_509 | CLF > CLW * | - | - |
| endo-xylogalacturonan hydrolase (EC 3.2.1.-) | - | CLC > CLW * | - |
| sterol glucosyltransferase (EC 2.4.1.173) | CLF < CLW * | - | - |
| anthocyanin 5-O-glucosyltransferase (EC 2.4.1.-) | CLF < CLW * | - | - |
| [inverting] exo-alpha-1,5-L-arabinanase (EC 3.2.1.-) | CLF > CLW ** | CLC > CLW * | - |
| isoamylase (EC 3.2.1.68) | CLF > CLW * | - | CLF > CLC * |
| flavonol L-rhamnosyltransferase (EC 2.4.1.159) | CLF < CLW * | - | - |
| xylan 1,4-beta-xylosidase (EC 3.2.1.37) | - | CLC > CLW * | - |
| mannan endo-beta-1,4-mannosidase (EC 3.2.1.78) | CLF > CLW * | - | - |
| alpha-1,6-mannanase (EC 3.2.1.101) | - | CLC > CLW * | - |
| UDP-beta-L-rhamnose: alpha-L-rhamnosyltr(...)_177 | CLF < CLW * | - | - |
| beta-1,3-xylosidase (EC 3.2.1.-) | CLF > CLW ** | CLC > CLW * | - |
| levan fructosyltransferase (EC 2.4.1.-) | CLF > CLW * | - | - |
| amylo-alpha-1,6-glucosidase (EC 3.2.1.33) | CLF > CLW * | - | CLF > CLC * |
| exo-alpha-1,5-L-arabinofuranosidase (EC 3.2.1.-) | CLF > CLW ** | CLC > CLW * | - |
| dodecaprenyl phosphate-beta-galacturonic(...)_293 | CLF < CLW * | - | - |
| prunasin beta-glucosidase (EC 3.2.1.118) | CLF > CLW ** | CLC > CLW * | - |
| mannosyl-oligosaccharide alpha-1,3-1,6-m(...)_435 | - | CLC > CLW * | - |
| galactoside alpha-1,3_1,4-L-fucosyltransferase (EC 2.4.1.65) | - | CLC > CLW * | - |
| glucan beta-1,3-glucosidase (EC 3.2.1.58) | CLF > CLW * | - | - |
| beta-N-acetylglucosaminide phosphorylases (EC 2.4.1.-) | - | CLC > CLW * | - |
| maltotriose-forming alpha-amylase (EC 3.2.1.116) | CLF > CLW * | - | CLF > CLC * |
| phosphatidylinositol alpha-mannosyltransferase (EC 2.4.1.57) | CLF < CLW * | CLC < CLW * | - |
| endo-beta-1,6-galactanase (EC 3.2.1.164) | CLF > CLW ** | CLC > CLW * | - |
| endo-beta-1,3-galactanase (EC 3.2.1.181) | CLF > CLW * | - | - |
| maltohexaose-forming alpha-amylase (EC 3.2.1.98) | CLF > CLW * | - | CLF > CLC * |
| D-galactosyl-beta-1,4-L-rhamnose phospho(...)_136 | CLF > CLW * | - | - |
| zeaxanthin glucosyltransferase (EC 2.4.1.-) | CLF < CLW * | - | - |
| diacylglycerol O-acyltransferase (EC 2.3.1.20) | CLF > CLW ** | CLC > CLW * | - |
| GDP-Man: Man3GlcNAc2-PP-dolichol_Man4Glc(...)_149 | CLF < CLW * | CLC < CLW * | - |
| glycoprotein alpha-1,3-L-fucosyltransferase (EC 2.4.1.214) | - | CLC > CLW * | - |
| alpha-L-iduronidase (EC 3.2.1.76) | CLF > CLW * | CLC > CLW * | - |
| maltopentaose-forming alpha-amylase (EC 3.2.1.-) | CLF > CLW * | - | CLF > CLC * |
| carboxylesterase (EC 3.1.1.1) | CLF > CLW ** | CLC > CLW * | - |
| feruloyl esterase (EC 3.1.1.73) | CLF > CLW ** | CLC > CLW * | - |
| trehalose-6-phosphate phosphorylase (EC 2.4.1.216) | - | CLC < CLW * | - |
| beta-1,4-mannosyl-glycoprotein beta-1,4-(...)_78 | CLF > CLW * | - | - |
| cycloisomaltooligosaccharide glucanotran(...)_275 | - | CLC > CLW * | - |
| phlorizin hydrolase (EC 3.2.1.62) | CLF > CLW ** | CLC > CLW * | - |
| endo-beta-1,4-glucanase _ cellulase (EC 3.2.1.4) | CLF > CLW * | - | - |
| lactase (EC 3.2.1.108) | CLF > CLW ** | CLC > CLW * | - |
| steryl beta-glucosidase (EC 3.2.1.104) | CLF > CLW * | - | - |
| UDP-GalNAc: N,N_-diacetylbacillosaminyl-(...)_188 | CLF < CLW * | CLC < CLW * | - |
| galacto-N-biose_lacto-N-biose phosphorylase (EC 2.4.1.-) | CLF > CLW * | - | - |
| UDP-GalNAc: GalNAc-PP-Und alpha-1,3-N-ac(...)_187 | CLF < CLW * | CLC < CLW * | - |
| alpha-xylosidase (EC 3.2.1.177) | CLF > CLW * | CLC > CLW * | - |
| beta-primeverosidase (EC 3.2.1.149) | CLF > CLW ** | CLC > CLW * | - |
| sucrose:fructan 6-fructosyltransferase (EC 2.4.1.10) | CLF > CLW * | - | - |
| fructan:fructan 6G-fructosyltransferase (EC 2.4.1.243) | CLF > CLW * | - | - |
| malto-oligosyltrehalose trehalohydrolase (EC 3.2.1.141) | CLF > CLW * | - | CLF > CLC * |
| vicianin hydrolase (EC 3.2.1.119) | CLF > CLW ** | CLC > CLW * | - |
| hydroxyisourate hydrolase (EC 3.-.-.-) | CLF > CLW ** | CLC > CLW * | - |
| beta-glucosidase (EC 3.2.1.21) | CLF > CLW ** | CLC > CLW ** | - |
| endo-beta-1,4-galactanase (EC 3.2.1.89) | CLF > CLW ** | CLC > CLW * | - |
| branching enzyme (EC 2.4.1.18) | CLF > CLW * | - | CLF > CLC * |
| tomatinase (EC 3.2.1.-) | CLF > CLW ** | CLC > CLW * | - |
| keratan-sulfate endo-1,4-beta-galactosidase (EC 3.2.1.103) | CLF > CLW * | - | - |
| alpha-L-fucosidase (EC 3.2.1.51) | CLF < CLW * | - | - |
| S-formylglutathione hydrolase (EC 3.1.2.12) | CLF > CLW ** | CLC > CLW * | - |
| cellulose beta-1,4-cellobiosidase (EC 3.2.1.91) | CLF > CLW * | - | - |
| GDP-Man: inositol-phosphorylceramide tra(...)_150 | CLF < CLW * | - | - |
| glucuronoarabinoxylan endo-beta-1,4-xylanase (EC 3.2.1.136) | CLF > CLW * | - | - |
| alpha-glucosidase (EC 3.2.1.20) | CLF > CLW ** | - | - |
| galactocerebrosidase (EC 3.2.1.46) | CLF > CLW ** | - | - |
| fructan beta-(2,6)-fructosidase_6-exohyd(...)_340 | CLF > CLW * | - | - |
| cinnamoyl esterase (EC 3.1.1.-) | CLF > CLW ** | CLC > CLW * | - |
| alpha-glucan lyase (EC 4.2.2.13) | CLF > CLW * | CLC > CLW * | - |
| UDP-Glc alpha-glucosyltransferase (EC 2.4.1.-) | CLF < CLW * | CLC < CLW * | - |
| N-acylsphingosine galactosyltransferase (EC 2.4.1.47) | CLF < CLW * | - | - |
| chitosanase (EC 3.2.1.132) | CLF > CLW * | - | - |
| kojibiose phosphorylase (EC 2.4.1.230) | - | CLC < CLW * | - |
| 2-O-alpha-glucopyranosylglycerol: phosph(...)_118 | - | CLC < CLW * | - |
| endo-1,3-beta-xylanase (EC 3.2.1.32) | CLF > CLW ** | CLC > CLW * | - |
| rhamnogalacturonase (EC 3.2.1.171) | - | CLC > CLW * | - |
| isoflavonoid 7-O-beta-apiosyl-beta-gluco(...)_391 | CLF > CLW ** | CLC > CLW * | - |
| xylosylprotein beta-4-galactosyltransferase (EC 2.4.1.133) | CLF < CLW ** | - | - |
| beta-2,6-fructan 6-levanbiohydrolase (EC 3.2.1.64) | CLF > CLW * | - | - |
| UDP-GlcNAc: ribostamycin alpha-N-acetylg(...)_208 | CLF < CLW * | CLC < CLW * | - |
| xylan alpha-1,2-glucuronidase (EC 3.2.1.131) | CLF > CLW * | CLC > CLW * | - |
| sucrose phosphorylase (EC 2.4.1.7) | CLF > CLW * | - | CLF > CLC * |
| malto-oligosyltrehalose synthase (EC 5.4.99.15) | CLF > CLW * | - | CLF > CLC * |
| cyclomaltodextrin glucanotransferase (EC 2.4.1.19) | CLF > CLW * | - | CLF > CLC * |
| endo-1,3(4)-beta-glucanase (EC 3.2.1.6) | CLF > CLW * | - | - |
| alpha-1,4-N-acetylglucosaminyltransferase (EC 2.4.1.-) | CLF < CLW * | - | - |
| trehalose synthase (EC 5.4.99.16) | CLF > CLW * | - | CLF > CLC * |
| glucan 1,4-beta-glucosidase (EC 3.2.1.74) | - | CLC > CLW * | - |
| k-carrageenase (EC 3.2.1.83) | CLF > CLW * | - | - |
| endoglycoceramidase (EC 3.2.1.123) | CLF > CLW * | - | - |
| alpha-glucosyltransferase (EC 2.4.1.52) | CLF < CLW * | CLC < CLW * | - |
| lipopolysaccharide alpha-1,3-galactosylt(...)_411 | CLF > CLW * | CLC > CLW * | - |
| [retaining] sucrose:sucrose 6-fructosylt(...)_220 | CLF > CLW * | - | - |
| sucrase-isomaltase (EC 3.2.1.48) (EC 3.2.1.10) | CLF > CLW * | CLC > CLW * | - |
| alpha-L-arabinopyranosidase (EC 3.2.1.-) | CLF > CLW * | - | - |
| alpha-amylase (EC 3.2.1.1) | CLF > CLW * | - | CLF > CLC * |
| beta-1,3-galactosyl-O-glycosyl-glycoprot(...)_66 | CLF > CLW * | - | - |
| endo-beta-1,3(4)-glucanase _ lichenase-l(...)_300 | - | CLC > CLW * | - |
| zeatin O-beta-xylosyltransferase (EC 2.4.2.40) | CLF < CLW * | - | - |
| isoprimeverose-producing oligoxyloglucan(...)_395 | - | CLC > CLW * | - |
| zeatin O-beta-glucosyltransferase (EC 2.4.1.203) | CLF < CLW * | - | - |
| alpha-1,4-mannosidase (EC 3.2.1.-) | CLF > CLW * | - | - |
| limonoid glucosyltransferase (EC 2.4.1.210) | CLF < CLW * | - | - |
| exo-1,3-1,4-glucanase (EC 3.2.1.-) | - | CLC > CLW * | - |
| [reducing end] beta-xylosidase (EC 3.2.1.-) | CLF > CLW * | - | - |
| DIMBOA beta-glucosidase (EC 3.2.1.182) | CLF > CLW ** | CLC > CLW * | - |
| lichenase _ endo-beta-1,3-1,4-glucanase (EC 3.2.1.73) | - | CLC > CLW * | - |
| beta-glucosylceramidase (EC 3.2.1.45) | CLF > CLW * | CLC > CLW * | - |
| oligosaccharide alpha-1,4-glucosyltransferase (EC 2.4.1.161) | CLF > CLW * | CLC > CLW * | - |
| endo-1,4-beta-xylanase (EC 3.2.1.8) | CLF > CLW ** | CLC > CLW * | - |
| maltogenic amylase (EC 3.2.1.133) | CLF > CLW * | - | CLF > CLC * |
| glucan endo-1,6-beta-glucosidase (EC 3.2.1.75) | CLF > CLW * | - | - |
| indole-3-acetate beta-glucosyltransferase (EC 2.4.1.121) | CLF < CLW * | - | - |
| alpha-1,2-mannosidase (EC 3.2.1.-) | CLF > CLW * | - | - |
| sucrose synthase (EC 2.4.1.13) | CLF < CLW * | CLC < CLW * | - |
| beta-glycosidase (EC 3.2.1.-) | CLF > CLW ** | CLC > CLW * | - |
| exopolygalacturonate lyase (EC 4.2.2.9) | CLF > CLW * | CLC > CLW * | - |
| 2-hydroxyacylsphingosine 1-beta-galactos(...)_119 | CLF < CLW * | - | - |
| licheninase (EC 3.2.1.73) | CLF > CLW * | - | - |
| alpha-L-arabinofuranosidase (EC 3.2.1.55) | CLF > CLW ** | CLC > CLW * | - |
| alpha-1,3-mannosidase (EC 3.2.1.-) | CLF > CLW * | - | - |
| glucosylceramidase (EC 3.2.1.45) | CLF > CLW * | - | - |
| trehalose phosphorylase (EC 2.4.1.64) | - | CLC < CLW * | - |
| homogalacturonan alpha-1,4-galacturonosy(...)_378 | CLF > CLW * | CLC > CLW * | - |
| alpha-1,6-mannosyltransferase (EC 2.4.1.-) | CLF < CLW * | - | - |
| arabinoxylan-specific endo-beta-1,4-xylanase (EC 3.2.1.-) | CLF > CLW * | - | - |
| coniferin beta-glucosidase (EC 3.2.1.126) | - | CLC > CLW * | - |
| beta-1,4-N-acetylglucosaminyltransferase (EC 2.4.1.-) | CLF < CLW ** | - | - |
| exo-beta-1,4-galactanase (EC 3.2.1.-) | CLF > CLW * | CLC > CLW * | - |
| glucan 1,3-beta-glucosidase (EC 3.2.1.58) | - | CLC > CLW * | - |
| trehalose 6-O-mycolyltransferase (EC 2.3.1.122) | CLF > CLW ** | CLC > CLW * | - |
| UDP-GlcA: xylan alpha-glucuronyltransferase (EC 2.4.1.-) | CLF > CLW * | CLC > CLW * | - |
| d-4,5-unsaturated beta-glucuronyl hydrolase (EC 3.2.1.-) | - | CLC > CLW * | - |
| endo-beta-1,4-xylanase (EC 3.2.1.8) | CLF > CLW ** | CLC > CLW * | - |
| ecdysteroid UDP-glucosyltransferase (EC 2.4.1.-) | CLF < CLW * | - | - |
| UDP-Gal: lactose_N-acetyl-lactosamine al(...)_182 | CLF < CLW * | - | - |
| neopullulanase (EC 3.2.1.135) | CLF > CLW * | - | CLF > CLC * |
| isomaltosyltransferase (EC 2.4.1.-) | CLF > CLW * | CLC > CLW * | - |
| alpha-mannosidase (EC 3.2.1.24) | CLF > CLW * | - | - |
| raffinose synthase (EC 2.4.1.82) | CLF > CLW ** | - | - |
| UDP-Gal: neolactotriaosylceramide beta-1(...)_183 | CLF < CLW ** | - | - |
| exo-polygalacturonosidase (EC 3.2.1.82) | - | CLC > CLW * | - |
| beta-glucuronidase (EC 3.2.1.31) | CLF > CLW * | CLC > CLW * | - |
| isopullulanase (EC 3.2.1.57) | CLF > CLW * | - | - |
| beta-1,3-mannanase (EC 3.2.1.-) | CLF > CLW * | - | - |
| sucrose-phosphate synthase (EC 2.4.1.14) | CLF < CLW * | CLC < CLW * | - |
| alpha-L-rhamnosidase (EC 3.2.1.40) | - | CLC > CLW * | - |
| nigerose phosphorylase (EC 2.4.1.279) | - | CLC < CLW * | - |
| xyloglucan-specific endo-beta-1,4-glucan(...)_520 | - | CLC > CLW * | - |
| endo-levanase (EC 3.2.1.65) | CLF > CLW * | - | - |
| invertase (EC 3.2.1.26) | CLF > CLW * | - | - |
| flavonol 3-O-glucosyltransferase (EC 2.4.1.91) | CLF < CLW * | - | - |
| UDP-Gal alpha-galactosyltransferase (EC 2.4.1.-) | CLF < CLW * | CLC < CLW * | - |
| N-acetyllactosamine synthase (EC 2.4.1.90) | CLF < CLW ** | - | - |
| trehalose-6-phosphate hydrolase (EC 3.2.1.93) | CLF > CLW * | - | CLF > CLC * |
| inositol 1-alpha-galactosyltransferase ((...)_385 | CLF > CLW * | CLC > CLW * | - |
| 6-phospho-beta-glucosidase (EC 3.2.1.86) | CLF > CLW * | CLC > CLW * | - |
| endo-1,3-beta-glucanase (EC 3.2.1.39) | CLF > CLW * | - | - |
| dolichyl-diphosphooligosaccharide芒鈧€(...)_294 | CLF > CLW * | - | - |
| glycogenin glucosyltransferase (EC 2.4.1.186) | CLF > CLW * | CLC > CLW * | - |
| alpha-glucuronidase (EC 3.2.1.139) | CLF > CLW ** | CLC > CLW * | - |
| unsaturated rhamnogalacturonyl hydrolase (EC 3.2.1.172) | - | CLC > CLW * | - |
| mannosyl-oligosaccharide alpha-1,2-manno(...)_434 | CLF > CLW * | - | - |
| amygdalin beta-glucosidase (EC 3.2.1.117) | CLF > CLW ** | CLC > CLW * | - |
| salicylic acid beta-glucosyltransferase (EC 2.4.1.-) | CLF < CLW * | - | - |
| N-acetyl-1-D-myo-inosityl-2-amino-2-deox(...)_160 | CLF > CLW * | CLC > CLW * | - |
| 3-deoxy-D-manno-octulosonic-acid hydrolase (EC 3.2.1.-) | CLF < CLW ** | - | - |
| ADP-dependent alpha-maltose-1-phosphate (...)_132 | CLF < CLW * | CLC < CLW * | - |
| trehalose phosphorylase (EC 2.4.1.231) | CLF < CLW * | CLC < CLW * | - |
| 6_-P-sucrose phosphorylase (EC 2.4.1.-) | CLF > CLW* | - | CLF > CLC * |
| GDP-Man: Man1GlcNAc2-PP-dolichol alpha-1(...)_147 | CLF < CLW * | CLC < CLW * | - |
| glucoamylase (EC 3.2.1.3) | CLF > CLW * | CLC > CLW * | - |
| beta-mannosidase (EC 3.2.1.25) | CLF > CLW * | CLC > CLW * | - |
| lacto-N-biose phosphorylase or galacto-N(...)_400 | CLF > CLW * | - | - |
| 4-alpha-glucanotransferase (EC 2.4.1.25) | CLF > CLW * | - | CLF > CLC * |
| chitin beta-1,6-glucanosyltransferase (EC 2.4.1.-) | CLF > CLW * | - | - |
| beta-fucosidase (EC 3.2.1.38) | CLF > CLW * | - | - |
| exo-polygalacturonate lyase (EC 4.2.2.9) | - | CLC > CLW * | - |
| endo-beta-1,4-galactosidase (EC 3.2.1.-) | CLF > CLW * | - | - |
| xyloglucanase (EC 3.2.1.151) | CLF > CLW * | - | - |
| beta-1,3-galactosidase (EC 3.2.1.-) | CLF > CLW * | CLC > CLW * | - |
| endoglucanase (EC 3.2.1.4) | CLF > CLW ** | CLC > CLW * | - |
| endo-inulinase (EC 3.2.1.7) | CLF > CLW * | - | - |
| exo-beta-1,4-glucanase (EC 3.2.1.74) | CLF > CLW ** | CLC > CLW * | - |
| oligo-alpha-glucosidase (EC 3.2.1.10) | CLF > CLW * | - | CLF > CLC * |
| ABA-specific beta-glucosidase (EC 3.2.1.175) | CLF > CLW ** | CLC > CLW * | - |
| alpha-2-O-mannosylglycerate hydrolase (EC 3.2.1.170) | - | CLC > CLW * | - |
| raucaffricine beta-glucosidase (EC 3.2.1.125) | CLF > CLW ** | CLC > CLW * | - |
| mannan transglycosylase (EC 2.4.1.-) | CLF > CLW * | - | - |
| fructan beta-(2,1)-fructosidase_1-exohyd(...)_339 | CLF > CLW * | - | - |
| diacetylchitobiose deacetylase (EC 3.5.1.-) | CLF > CLW * | CLC > CLW * | - |
| thioglucosidase (EC 3.2.1.147) | CLF > CLW ** | CLC > CLW * | - |
| amylosucrase (EC 2.4.1.4) | CLF > CLW * | - | CLF > CLC * |
| xyloglucan:xyloglucosyltransferase (EC 2.4.1.207) | CLF > CLW * | - | - |
| UDP-Glc: chalcone 4芒鈧?O-beta-glucos(...)_197 | CLF < CLW * | - | - |
| arabinanase (EC 3.2.1.99) | CLF > CLW ** | CLC > CLW * | - |
| sulfoquinovosidase (EC 3.2.1.-) | CLF > CLW * | CLC > CLW * | - |
| lipopolysaccharide glucosyltransferase 1 (EC 2.4.1.58) | CLF > CLW * | CLC > CLW * | - |
| galactoside alpha-1,3-L-fucosyltransferase (EC 2.4.1.152) | - | CLC > CLW * | - |
| exo-polygalacturonase (EC 3.2.1.67) | - | CLC > CLW * | - |
| glycogen or starch phosphorylase (EC 2.4.1.1) | CLF > CLW * | CLC > CLW * | - |
| UDP-GlcNAc: 2-deoxystreptamine alpha-N-a(...)_204 | CLF < CLW * | CLC < CLW * | - |
| exo-inulinase (EC 3.2.1.80) | CLF > CLW * | - | - |
| anthocyanidin 3-O-glucosyltransferase (EC 2.4.1.115) | CLF < CLW * | - | - |
| dextranase (EC 3.2.1.11) | CLF > CLW * | CLC > CLW * | - |
| endo-alpha-N-acetylgalactosaminidase (EC 3.2.1.97) | CLF > CLW * | - | - |
| digalactosyldiacylglycerol synthase (EC 2.4.1.141) | CLF < CLW * | CLC < CLW * | - |
| fructan:fructan 1-fructosyltransferase (EC 2.4.1.100) | CLF > CLW * | - | - |
| mannosyl-oligosaccharide alpha-1,6-mannosidase (EC 3.2.1.-) | CLF > CLW * | - | - |
| amylomaltase or 4-alpha-glucanotransferase (EC 2.4.1.25) | CLF > CLW * | - | - |
| beta-galactosidase (EC 3.2.1.23) | CLF > CLW ** | CLC > CLW ** | - |
| maltose phosphorylase (EC 2.4.1.8) | - | CLC < CLW * | - |
| trans-sialidase (EC 2.4.1.-) | CLF < CLW ** | - | - |
| xylanase (EC 3.2.1.8) | CLF > CLW ** | CLC > CLW * | - |
| exo-alpha-1,6-mannosidase (EC 3.2.1.-) | CLF > CLW * | - | - |
| pectate lyase (EC 4.2.2.2) | - | CLC > CLW * | - |
| sinapate 1-glucosyltransferase (EC 2.4.1.120) | CLF < CLW * | - | - |
| beta-L-arabinofuranosidase (EC 3.2.1.185) | CLF > CLW ** | CLC > CLW * | CLF > CLC * |
| beta-D-fucosidase (EC 3.2.1.38) | CLF > CLW ** | CLC > CLW * | - |
| glucodextranase (EC 3.2.1.70) | CLF > CLW * | - | CLF > CLC * |
| lipopolysaccharide N-acetylglucosaminylt(...)_413 | CLF < CLW * | - | - |
| mycothiol S-conjugate amidase (EC 3.5.1.-) | CLF > CLW * | CLC > CLW * | - |
| galactan 1,3-beta-galactosidase (EC 3.2.1.145) | CLF > CLW ** | CLC > CLW * | - |
| undecaprenyl phosphate-alpha-L-Ara4N: 4-(...)_508 | CLF < CLW * | - | - |
| lysozyme (EC 3.2.1.17) | - | CLC < CLW * | - |
| 3-C-carboxy-5-deoxy-L-xylose (aceric aci(...)_122 | CLF > CLW ** | CLC > CLW * | CLF > CLC * |
| isomaltulose synthase (EC 5.4.99.11) | CLF > CLW * | - | CLF > CLC * |
| xylan endotransglycosylase (EC 2.4.2.-) | CLF > CLW ** | CLC > CLW * | - |
| beta-N-acetylglucosaminyl-glycopeptide b(...)_92 | CLF < CLW ** | - | - |
| chitinase (EC 3.2.1.14) | CLF < CLW * | - | - |
| dextran 1,6-alpha-isomaltotriosidase (EC 3.2.1.95) | CLF > CLW * | - | - |
| xyloglucan-specific endo-beta-1,4-glucanase (EC 3.2.1.151) | CLF > CLW * | - | - |
| mannosyl-1-phosphodiester alpha-1,P-mannosidase (EC 3.2.1.-) | CLF > CLW * | - | - |
| alpha-glucosyl-1,2-beta-galactosyl-L-hyd(...)_49 | - | CLC < CLW * | - |
| alpha-1,4-glucan: phosphate alpha-maltos(...)_23 | CLF > CLW * | - | CLF > CLC * |
| mannosyl-oligosaccharide alpha-1,3-mannosidase (EC 3.2.1.-) | CLF > CLW * | - | - |
| beta-xylosidase (EC 3.2.1.37) | CLF > CLW ** | CLC > CLW * | - |
| 3-O-alpha-glucopyranosyl-L-rhamnose phos(...)_123 | - | CLC < CLW * | - |
| anthocyanin 3-O-galactosyltransferase (EC 2.4.1.-) | CLF < CLW * | - | - |
| UDP-GlcA:arabinogalactan beta-glucuronos(...)_202 | CLF > CLW * | - | - |
| 1,2-diacylglycerol 3-glucosyltransferase (EC 2.4.1.157) | CLF < CLW * | CLC < CLW * | - |
| NDP-Glc: alpha-glucose alpha-glucosyltra(...)_171 | CLF < CLW * | CLC < CLW * | - |
| L-Rhalpha-alpha-1,4-GlcA alpha-L-rhamnoh(...)_153 | CLF > CLW * | CLC > CLW * | - |
| UDP-GlcA alpha-glucuronyltransferase (EC 2.4.1.-) | CLF < CLW * | CLC < CLW * | - |
